# Development of Albumin Nanocarriers for Enhanced Curcumin Delivery and In Vitro Anticancer Activity in Colon Cancer Cells

**DOI:** 10.3390/ph19060872

**Published:** 2026-05-30

**Authors:** Aftab Ahmad, Darshana Bagwe, Shagufta Khan, Chetna Dhone, Shilpa Padhare, Anwar A. Alghamdi, Shah Alam Khan

**Affiliations:** 1 Health Information Technology Department, The Applied College, King Abdulaziz University, Jeddah 21589, Saudi Arabia; abdulsalam@kau.edu.sa (A.A.); nloalgamdi7@kau.edu.sa (A.A.A.); 2 Pharmacovigilance and Medication Safety Unit, Center of Research Excellence for Drug Research and Pharmaceutical Industries, King Abdulaziz University, Jeddah 21589, Saudi Arabia; 3 Department of Pharmaceutics, Institute of Pharmaceutical Education and Research, Wardha Borgaon (Meghe), Wardha 442 001, Maharashtra, India; globalalldata@gmail.com (D.B.); chetnadhone2000@gmail.com (C.D.); svpadhare@gmail.com (S.P.); 4 Department of Pharmaceutical Chemistry, College of Pharmacy, National University of Science and Technology, Bousher, Muscat PC 130, Oman; shahalam@nu.edu.om

**Keywords:** curcumin, albumin nanocarriers, bovine serum albumin, pH-dependent drug delivery, colon cancer, Colo-205 cells, protein-based nanoparticles, controlled release, anticancer nanomedicine

## Abstract

**Objectives:** Curcumin possesses well-documented anticancer activity; however, its clinical translation is hindered by poor aqueous solubility and limited bioavailability. The present study aimed to engineer pH-dependent bovine serum albumin (BSA)–based nanocarriers for curcumin delivery and to evaluate their physicochemical characteristics, controlled release behavior under gastrointestinal pH conditions, and in vitro anticancer efficacy against the human colon cancer cell line Colo-205. **Methods:** Curcumin-loaded bovine serum albumin nanoparticles (Cu-BSA-NPs) were fabricated using a desolvation technique followed by chemical crosslinking. Particle size, zeta potential, and polydispersity index (PDI) were assessed by dynamic light scattering. Morphology was examined using scanning electron microscopy (SEM), while structural and thermal properties were evaluated by Fourier-transform infrared spectroscopy (FTIR) and differential scanning calorimetry (DSC). Drug loading capacity and entrapment efficiency were quantified spectrophotometrically. In vitro drug release was investigated using a gastrointestinal pH-transition model (pH 1.2, 6.8, and 7.4). Cytotoxic activity was assessed using the sulforhodamine B (SRB) assay on Colo-205 cells. **Results:** The engineered Cu-BSA-NPs exhibited particle sizes ranging from 96.7 ± 10.5 to 126.4 ± 35.8 nm, with PDI values between 0.289 and 0.581 and zeta potentials from −18.2 ± 1.01 to −34 ± 1.0 mV, indicating nanoscale dimensions and moderate colloidal stability. SEM analysis revealed spherical nanoparticles with smooth surfaces and uniform morphology. Entrapment efficiency ranged from 6.59 ± 1.11% to 52.98 ± 0.65%, while drug loading efficiency varied between 1.308 ± 0.206% and 16.744 ± 0.266%. In vitro release studies demonstrated minimal drug release under acidic (pH 1.2) and near-neutral (pH 6.8) conditions, followed by significantly enhanced release at pH 7.4, confirming pH-dependent behavior of the albumin matrix. Cytotoxicity studies showed significant antiproliferative activity against Colo-205 human colon cancer cells. **Conclusions:** The findings demonstrate successful engineering of albumin-based nanocarriers capable of modulating curcumin release under physiologically relevant pH conditions and enhancing in vitro anticancer activity. Although limited to in vitro evaluation, this study highlights the potential of protein-based nanoplatforms as adaptable delivery systems for colon cancer therapy. Further in vivo investigations are warranted to validate their translational and therapeutic potential.

## 1. Introduction

Colorectal cancer is among the most frequently diagnosed malignancies globally and remains a major contributor to cancer-associated mortality, along with breast, prostate, and lung cancers [1,2]. The development of colon cancer is influenced by multiple risk factors, including family history, genetic alterations, obesity, sex, advancing age, and comorbid conditions such as diabetes and inflammatory bowel disease [3,4]. Colon cancer primarily develops as a result of alterations in the normal colonic epithelium, leading to abnormal cellular proliferation in the intestinal lining and the formation of adenomatous polyps [3]. Common therapeutic approaches for colon cancer include surgical resection, radiotherapy, cryosurgery, and systemic chemotherapy. Among these, conventional chemotherapy remains one of the most widely used approaches; however, due to its lack of selectivity, chemotherapeutic agents are often associated with significant adverse effects [4]. The development of nanoparticles (NPs) has enabled site-specific drug release through targeted drug delivery systems (TDDS), thereby improving therapeutic efficacy while reducing systemic toxicity in the treatment of colon cancer. Targeted drug delivery systems also help minimize the adverse effects of chemotherapy by limiting drug exposure to healthy tissues, as nanoparticles are designed to preferentially accumulate in cancerous cells [4,5].

Therapeutic nanoparticles have been widely explored for the management of various diseases [6]. In particular, protein-based nanoparticles have gained considerable attention in the field of nanomedicine because of their distinctive biological properties and broad applicability in drug delivery, diagnostic imaging, and therapeutic interventions. These protein-derived nanocarriers have been designed to facilitate targeted drug delivery, with the goal of enhancing therapeutic efficacy while reducing off-target effects and treatment-related toxicity. These nanocarrier systems exhibit high loading versatility, allowing efficient encapsulation of a wide range of bioactive molecules, including proteins, peptides, nucleic acids and small-molecule drugs. In addition to enhancing drug stability by protecting therapeutic agents from premature degradation, nanoparticles can facilitate controlled and site-specific release of the payload [6]. Moreover, engineered nanoparticles may enhance therapeutic outcomes owing to their favorable biocompatibility and capacity to interact with biological systems in a manner that resembles endogenous cellular processes. Consequently, protein-based nanocarriers represent a promising strategy for supporting the development of personalized treatment approaches and improving overall patient care [7]. Solanki et al. recently explored bovine serum albumin (BSA) nanoparticles as nanocarrier systems for anticancer drug delivery applications [8]. BSA, a protein-based carrier, is water-soluble, biocompatible, biodegradable, non-toxic, and exhibits low immunogenicity, in addition to possessing a high drug-binding capacity. These characteristics render it a promising platform for the design and development of effective anticancer nanoformulations. Albumin-derived nanocarriers have therefore gained considerable attention as promising delivery systems, owing to their inherent biological compatibility and their ability to utilize endogenous albumin transport pathways for enhanced tumor targeting. Tumor cells and tumor-associated endothelial cells are reported to overexpress albumin-binding proteins, including glycoprotein 60 (gp60) and secreted protein acidic and rich in cysteine (SPARC). These proteins promote albumin transcytosis and facilitate its accumulation within the tumor microenvironment. Binding of albumin to gp60 receptors on endothelial cells can activate caveolae-mediated transport pathways, thereby enhancing the intracellular delivery of albumin-associated therapeutic cargos [9,10,11,12]. It should be noted that the present study does not experimentally investigate receptor-mediated uptake or active targeting mechanisms; these aspects are discussed solely with reference to previously published findings. Among naturally derived plant-based anticancer agents, curcumin has been extensively studied for its ability to interact with diverse intracellular targets, including proteins and nucleic acids. Existing evidence indicates that curcumin modulates multiple molecular signaling cascades, notably nuclear factor kappa-B (NF-κB), tumor protein p53, and activator protein-1 (AP-1), thereby influencing tumor cell proliferation, inflammatory responses, and apoptotic processes [13]. Curcumin belongs to the class of linear diarylheptanoids, characterized by a seven-carbon linker connecting two aromatic rings. Structural variations within this class, including functional group substitution and conjugation patterns, have been reported to influence physicochemical properties such as solubility, stability, and biological activity [14]. Despite its promising pharmacological profile, the clinical application of curcumin remains constrained by its hydrophobicity, limited aqueous solubility, poor oral bioavailability, and instability under physiological conditions [15]. Moreover, inefficient gastrointestinal absorption and extensive first-pass metabolism further reduce its systemic exposure and tissue distribution [16]. Natural bioenhancers, such as piperine, have been reported to enhance the bioavailability of curcumin by inhibiting drug-metabolizing enzymes, including cytochrome P450 3A isoforms, and by reducing intestinal and hepatic glucuronidation. In addition, piperine has been shown to modulate ATP-binding cassette (ABC) transporters, such as P-glycoprotein and related multidrug resistance proteins, thereby decreasing drug efflux and increasing intracellular retention of co-administered therapeutic agents [17]. Piperine was incorporated as a natural bioenhancer to improve the pharmacokinetic performance of curcumin.

Although albumin-based nanoparticles have been widely investigated for curcumin delivery, many reported systems rely on additional pH-sensitive polymer coatings or chemically modified carriers to achieve targeted drug release. In contrast, the present study explores Cu-BSA-NPs in which the observed pH-dependent release behavior arises primarily from the intrinsic physicochemical characteristics of the albumin matrix. The use of EDC as a zero-length crosslinking agent stabilizes the protein network through amide bond formation while preserving the inherent pH-dependent conformational flexibility of albumin. Furthermore, the desolvation-based preparation method [18] employed in this study is relatively simple, cost-effective, and potentially scalable, making it suitable for practical formulation development. This formulation strategy therefore enables pH-dependent drug release without requiring additional pH-sensitive polymers while maintaining a straightforward and scalable preparation approach.

Therefore, the present study was designed to develop Cu-BSA-NPs and to assess their in vitro cytotoxic potential against the human colon cancer cell line Colo-205.

## 2. Results

### 2.1. Apparent Solubility Study

Curcumin exhibited extremely low intrinsic solubility in all tested media, reaffirming its well-recognized formulation limitations. Encapsulation within bovine serum albumin nanoparticles resulted in a marked and consistent increase in solubility under all experimental conditions. In aqueous medium, the apparent solubility of curcumin increased substantially, with enhancement ratios ranging from approximately 31.11 ± 12.04 fold to nearly 280.37 ± 62.86 fold relative to the native compound, depending on the formulation. These findings demonstrate the effectiveness of albumin-based nanoencapsulation in improving curcumin dispersion in water. Under acidic (pH 1.2) and near-neutral (pH 6.8) conditions, solubility enhancement was moderate, ranging from approximately 11.31 ± 1.33 to 52.48 ± 5.31 fold and 14.22 ± 1.12 to 51.66 ± 12.48 fold, respectively. A more pronounced increase was observed at alkaline pH (7.4), where curcumin solubility improved by up to approximately 324.83 ± 57.79 fold. Among the tested formulations, batch AC6 consistently exhibited the greatest solubility enhancement across all media (Table 1).

Statistical analysis using one-way ANOVA revealed significant differences in curcumin solubility among the formulations in all tested media, including water (F = 13.40, *p* < 0.0001), pH 1.2 (F = 65.15, *p* < 0.0001), pH 6.8 (F = 44.33, *p* < 0.0001), and pH 7.4 (F = 41.62, *p* < 0.0001). Tukey–Kramer post hoc analysis demonstrated that several nanoparticle formulations exhibited significantly higher solubility compared with native curcumin. In water, formulations AC2, AC4, AC6, and AC7 showed significant enhancement, whereas in pH 1.2 and pH 6.8 media, all nanoparticle formulations displayed significantly improved solubility relative to native curcumin (*p* < 0.05). In pH 7.4 medium, formulations AC2, AC4, AC5, AC6, and AC7 exhibited statistically significant increases in solubility compared with native curcumin, with AC6 showing the greatest enhancement under alkaline conditions.

The substantial increase in apparent solubility compared to native curcumin confirms the effectiveness of the nanoparticulate system in maintaining the drug in a dispersed and stabilized state.

### 2.2. Encapsulation and Drug Loading Efficiency

The entrapment efficiency (EE%) and drug loading capacity of Cu-BSA-NPs were markedly affected by formulation variables, particularly the relative proportions of bovine serum albumin, curcumin, and the crosslinking agent EDC (Table 2). Variations in these components resulted in measurable differences in encapsulation performance across the prepared batches.

Formulations containing lower amounts of albumin and curcumin, such as AC1, demonstrated limited entrapment efficiency (6.59 ± 1.11%), suggesting that an insufficient protein matrix was available to effectively incorporate the drug. This finding emphasizes the importance of adequate albumin content in establishing a stable nanoparticle framework capable of retaining curcumin within its structure. A clear relationship between the BSA-to-curcumin ratio and encapsulation performance was observed across the prepared batches. Formulations prepared at a 2:1 BSA:Curcumin ratio consistently achieved higher entrapment efficiencies compared with those containing equal proportions of drug and polymer. Among all batches, AC6 exhibited the highest entrapment efficiency (52.98 ± 0.65%). This improved performance may be attributed to the optimized combination of increased albumin content (400 mg), moderate curcumin loading (200 mg), and a lower crosslinker concentration (0.5 mg EDC). Such a balanced composition likely provided sufficient binding sites within the albumin matrix while preserving structural flexibility for efficient drug incorporation.

A similar trend was observed for drug loading efficiency, which improved with an optimized carrier-to-drug ratio and lower crosslinking density. Loading efficiency varied considerably among the formulations, with AC6 again demonstrating the highest value (16.74 ± 0.27%). In contrast, batches containing lower curcumin amounts (e.g., AC2) or relatively higher crosslinker levels showed reduced loading efficiency. This suggests that limited drug availability or excessive matrix rigidity may have hindered effective drug entrapment.

Increasing the curcumin content beyond the optimal level, as observed in formulations AC3, AC5, and AC7, did not produce proportional improvements in entrapment efficiency or drug-loading capacity. This non-linear increase in apparent solubility and encapsulation efficiency with higher curcumin concentrations may be attributed to saturation of the available drug-binding and accommodation sites within the BSA matrix. Once the carrier reaches its effective loading capacity, excess curcumin is insufficiently incorporated into the protein network and may instead aggregate or precipitate, thereby reducing the fraction of drug effectively dispersed or encapsulated.

Similarly, higher crosslinker concentrations, particularly ≥1.25 mg EDC, appear to decrease the internal free volume of the protein network, restricting drug accommodation within the nanoparticle matrix. These findings suggest that an optimal balance between protein concentration and crosslinking density is essential for maximizing curcumin incorporation into BSA nanoparticles.

### 2.3. Particle Size and Zeta Potential Study

The mean particle size of the Cu-BSA-NPs ranged from 96.7 ± 10.5 nm (AC1) to 126.4 ± 35.8 nm (AC7), confirming that all batches were successfully prepared within the nanoscale range (Table 3). A gradual increase in particle size was observed with variations in formulation composition, particularly in formulations containing higher albumin content and lower crosslinker concentration.

The polydispersity index (PDI) values ranged from 0.256 (AC2) to 0.581 (AC1). Formulations AC2 and AC6 demonstrated relatively low PDI values (≤0.30), indicating a narrower size distribution and improved homogeneity. In contrast, higher PDI values observed in AC1 and AC7 suggest increased heterogeneity, potentially resulting from suboptimal formulation parameters or partial aggregation during nanoparticle formation. Overall, the PDI findings indicate that careful optimization of formulation variables contributed to better control over particle size distribution.

Zeta potential analysis revealed a negative surface charge for all formulations, with values ranging from −18.2 ± 1.01 mV (AC1) to −34.0 ± 1 mV (AC7). The progressive increase in negative charge may be associated with greater surface exposure of ionizable functional groups derived from bovine serum albumin and curcumin. Notably, AC6 exhibited a zeta potential of approximately −30 ± 2 mV, a value generally regarded as indicative of adequate colloidal stability, suggesting a lower tendency for particle aggregation under the tested conditions. The narrow size distribution and negative surface charge indicate good colloidal stability and suggest suitability for efficient cellular interaction.

### 2.4. FTIR Spectroscopy

**Figure 1:**
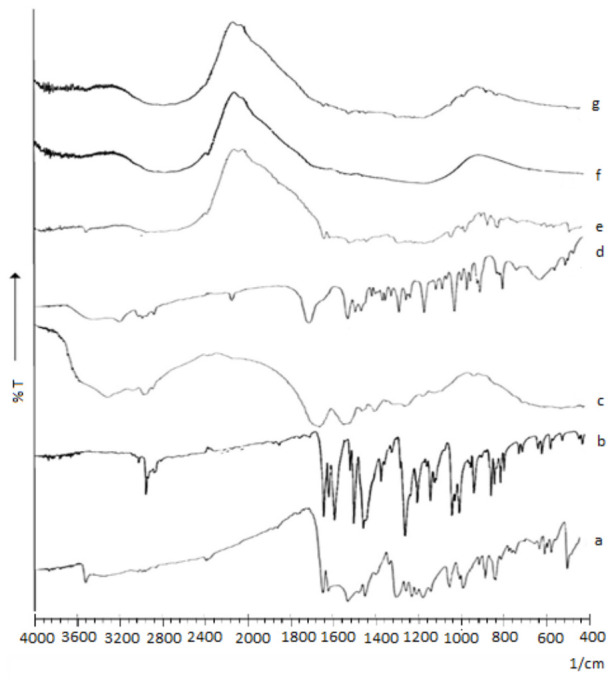
FTIR spectra of (**a**) Curcumin, (**b**) Piperine, (**c**) Bovine serum albumin (BSA), (**d**) EDC·HCl, (**e**) The physical mixture of curcumin, piperine, and BSA, (**f**) Unloaded BSA nanoparticles, and (**g**) Curcumin-loaded BSA nanoparticles (Cu-BSA-NPs).

The FTIR spectra revealed characteristic absorption peaks at 1280.65 cm^−1^ (aromatic –O–CH_3_ stretching), 1625.88 cm^−1^ (C=O stretching), 1232.43 cm^−1^ (–O stretching), and 1506.3 cm^−1^ (C–O stretching), confirming the presence of curcumin within the nanoparticle formulations (Figure 1). Bands observed in the region of 1200–1300 cm^−1^, corresponding to C–N and N–H stretching vibrations (Figure 1g), suggest successful crosslinking between BSA and EDC·HCl. In the FTIR spectra of the drug–excipient physical mixture and the curcumin-loaded nanoparticles, the principal characteristic peaks remained intact without significant shifts or disappearance. This indicates the absence of chemical incompatibility and confirms that the functional groups of curcumin and piperine were preserved during nanoparticle preparation. These findings confirm successful incorporation of curcumin within the BSA matrix without chemical degradation, suggesting physical entrapment rather than chemical modification.

### 2.5. Differential Scanning Calorimetric Study

**Figure 2:**
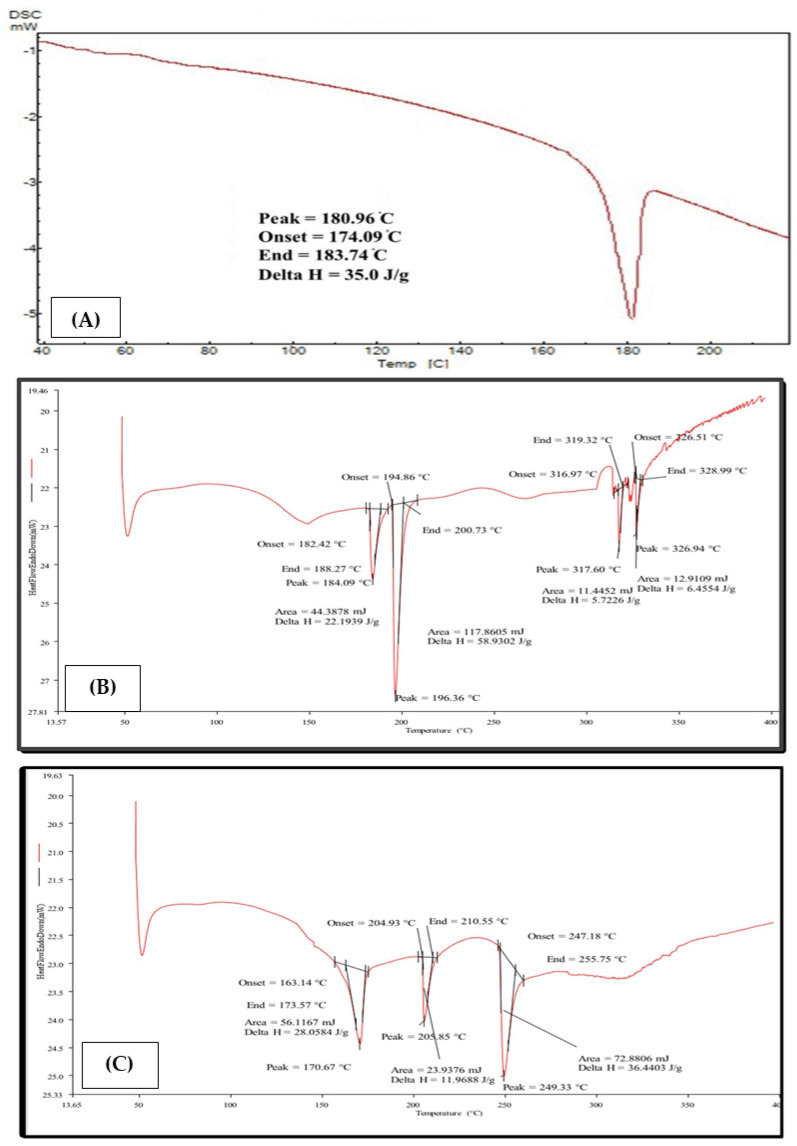
DSC thermograms of (**A**) Pure Curcumin, (**B**) Unloaded BSA nanoparticles, and (**C**) Cu-BSA-NPs.

The DSC thermogram of curcumin exhibited a distinct endothermic peak at 180.96 °C, corresponding to its characteristic melting point. The thermogram of unloaded BSA nanoparticles displayed a transition peak around 52 °C, attributed to thermal changes associated with the protein matrix. In the case of Cu-BSA-NPs, a sharp endothermic peak was observed at 170.67 °C, indicating the presence of curcumin within the nanoparticle system, although with a slight shift in melting temperature compared to the pure drug. This shift may suggest partial interaction or reduced crystallinity of curcumin within the albumin matrix. This shift may suggest partial interaction or reduced crystallinity of curcumin within the albumin matrix. The DSC profile of BSA exhibited multiple thermal transitions, reflecting structural changes within the protein. The lower-temperature transition is likely associated with disruption of primary structural interactions, whereas transitions at higher temperatures correspond to alterations in secondary and tertiary conformations (Figure 2).

### 2.6. Morphological Evaluation by Scanning Electron Microscopy (SEM)

**Figure 3:**
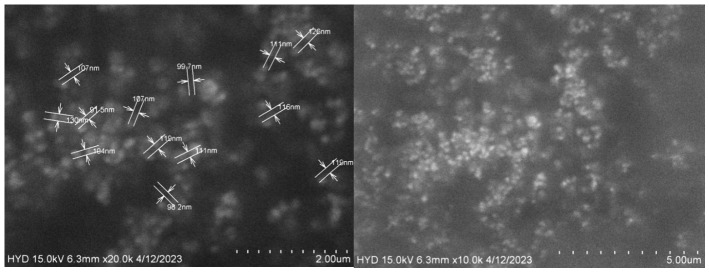
Scanning electron micrograph of Cur-BSA-NPs.

The morphological assessment of Cu3-BSA-NPs was performed using scanning electron microscopy (SEM). SEM of the NPs shows nearly discrete monodispers spherical particles with sizes ranging from 91.5 to 130 nm (Figure 3).

### 2.7. In Vitro Profile of Drug Release

**Figure 4:**
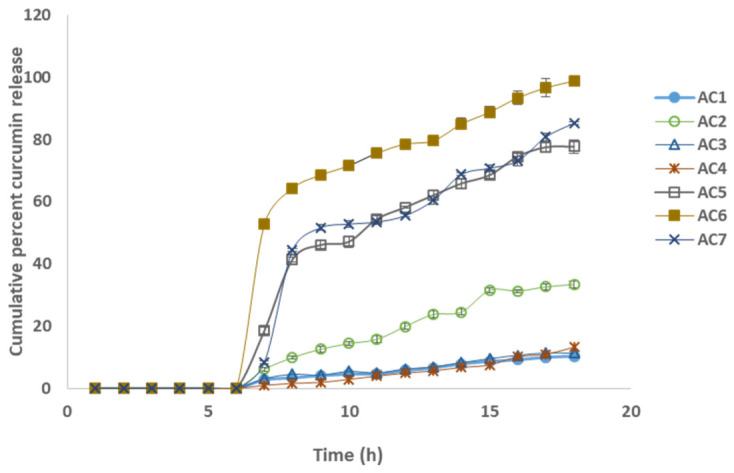
In vitro release profile of curcumin from Cu-BSA-NPs (AC1–AC7) under sequential pH conditions simulating gastrointestinal transit.

The in vitro release kinetics of curcumin from Cu-BSA-NPs (AC1–AC7) were evaluated under sequential pH conditions simulating gastrointestinal transit (pH 1.2 for 2 h, pH 6.8 for 4 h, followed by pH 7.4 up to 18 h), and the cumulative percentage drug release is shown in Figure 4.

Negligible curcumin release was observed from all formulations during the initial acidic phase (pH 1.2), indicating effective retention of the drug within the albumin matrix under gastric conditions. Similarly, only minimal release occurred during exposure to pH 6.8, suggesting limited drug diffusion under near-neutral conditions. This restricted release in the early phases reflects the compact nature of the albumin nanoparticle matrix under acidic and near-neutral environments. Drug release was minimal at acidic and near-neutral pH and increased significantly at pH 7.4. The human colon typically exhibits near-neutral to slightly alkaline pH conditions (approximately pH 6.8–7.5). The enhanced drug release observed at pH 7.4 therefore suggests that the albumin matrix may permit increased drug diffusion under conditions resembling the colonic environment.

A marked increase in curcumin release was observed upon transition to pH 7.4. The extent and rate of release varied significantly among the formulations, reflecting differences in formulation composition. Among all batches, AC6 exhibited the highest cumulative drug release, reaching nearly complete release (~100%) by the end of the study period. This formulation also demonstrated a relatively rapid release onset at pH 7.4, followed by sustained release over time. Formulations AC5 and AC7 showed moderate release profiles, with cumulative drug release reaching approximately 75–85% by the end of the study. In contrast, AC2 displayed limited release, achieving only about 30–35% cumulative release, while AC1, AC3, and AC4 showed minimal overall drug release (<15%), indicating suboptimal release behavior (Figure 4).

The increased drug release at pH 7.4 suggests that the formulation may favor drug release under conditions resembling the colonic environment.

The in vitro release data obtained between 7 and 18 h were subjected to kinetic modeling. Among the evaluated formulations, AC6 exhibited the best fit to the Higuchi model, with a coefficient of determination (R^2^) of 0.9543, suggesting that drug release was predominantly governed by diffusion. Furthermore, analysis using the Korsmeyer-Peppas model yielded a release exponent (*n*) value of 0.241 (Table 4), consistent with a Fickian diffusion mechanism as the principal mode of drug release from the nanoparticle system.

### 2.8. Stability Study

CuP-BSA-NPs exhibited no significant changes in drug loading or percentage entrapment efficiency over the six-month storage period. Statistical analysis using the Student’s t-test demonstrated that the differences observed were not statistically significant (*p* > 0.05), indicating that the formulation remained stable under the tested storage conditions (Table 5). The absence of significant changes over six months indicates good physical and chemical stability of the nanoparticle formulation.

### 2.9. Colo-205 Human Cell Line Study

**Figure 5:**
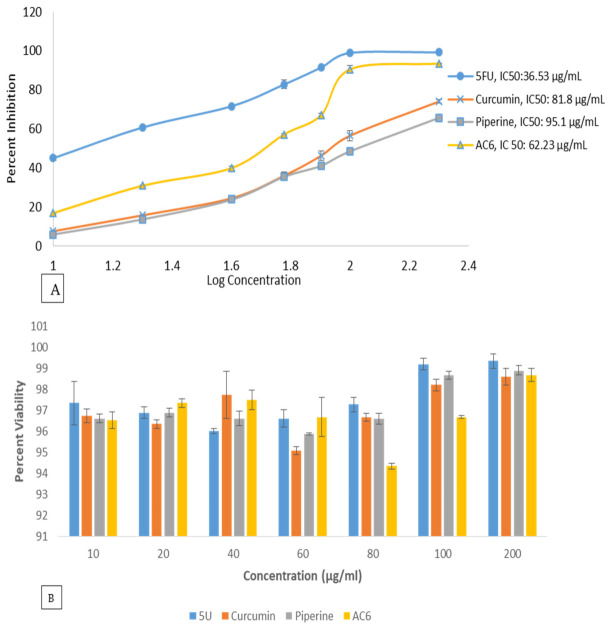
(**A**) In vitro cytotoxic activity of AC6, free curcumin, free piperine, and the reference anticancer drug 5-fluorouracil against the human colon cancer cell line Colo-205 after 48 h of treatment, as determined using the SRB assay. All tested samples exhibited concentration-dependent cytotoxicity, with AC6 showing enhanced activity compared with the free compounds. (**B**) Percentage cell viability of the non-cancerous human colon epithelial cell line CCD 841 following treatment with AC6, free curcumin, free piperine, and 5-fluorouracil. High cell viability was maintained across the tested concentration range, indicating minimal cytotoxicity toward normal cells under the experimental conditions. Data are presented as mean ± standard deviation (SD), *n* = 3.

The in vitro cytotoxic activity of the optimized AC6 was evaluated against the Colo-205 human colon cancer cell line and compared with free curcumin, free piperine, and the reference anticancer drug 5-fluorouracil. All tested samples produced a concentration-dependent reduction in cell viability (Supplementary Materials). The reference drug 5-fluorouracil exhibited the highest cytotoxic potency, with an IC_50_ value of 36.53 µg/mL. Among the tested formulations, AC6 showed greater cytotoxic activity than the free compounds, with an IC_50_ value of 62.23 µg/mL, compared with 81.8 µg/mL for free curcumin and 95.1 µg/mL for free piperine (Supplementary Materials). At 100 µg/mL, AC6 produced 90.5 ± 0.1% growth inhibition, whereas free curcumin and free piperine produced 56.47 ± 1.8% and 48.45 ± 2.6% inhibition, respectively. These findings indicate that incorporation of curcumin into the BSA nanoparticle system enhanced its in vitro anticancer activity against Colo-205 cells. This improvement may be associated with improved aqueous dispersibility and physicochemical stabilization of curcumin within the nanoparticle system under the tested in vitro conditions (Figure 5A).

### 2.10. Cytotoxicity on Non-Cancerous Cells

The cytotoxicity of the test samples was further evaluated in the non-cancerous CCD 841 human colon epithelial cell line to assess the safety profile of the formulation. As shown in Figure 5B, all tested samples maintained high cell viability across the evaluated concentration range of 10–200 µg/mL, indicating minimal cytotoxic effects on normal colon epithelial cells.

The reference drug, 5-fluorouracil, exhibited negligible cytotoxicity under the tested conditions, with cell viability remaining above 90% at all concentrations (Supplementary Materials). Similarly, free curcumin and free piperine showed minimal toxicity, with only a slight reduction in cell viability observed at higher concentrations. The nanoparticle formulation AC6 produced a modest decrease in cell viability compared with the free compounds; however, viability remained above 90% across the entire concentration range, including the highest concentration tested.

These results suggest that AC6 enhances cytotoxic activity against Colo-205 cancer cells while maintaining relatively low toxicity toward non-cancerous colon epithelial cells under in vitro conditions. This indicates a favorable preliminary safety profile and supports the potential selectivity of the Cu-BSA-NPs.

**Figure 6:**
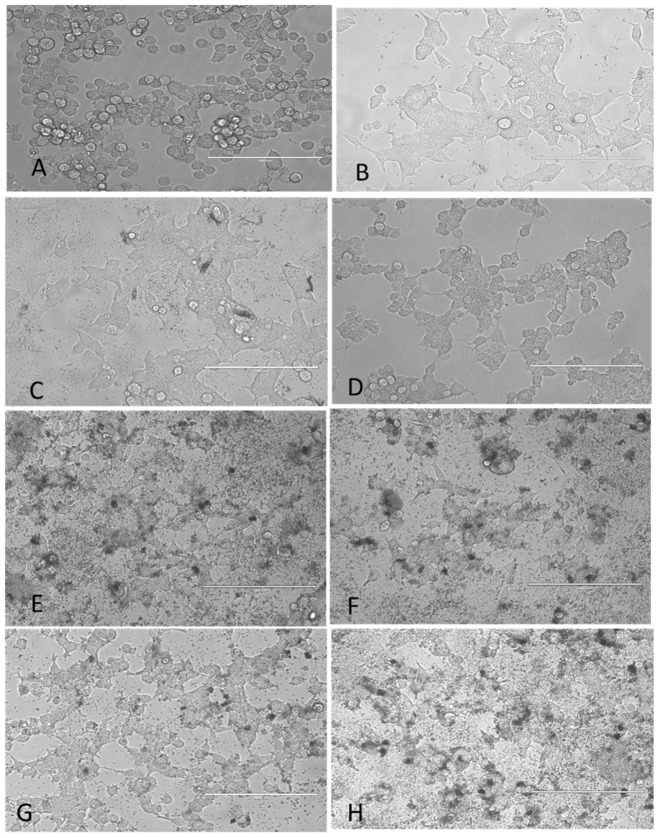
Phase-contrast microscopic images showing the morphological responses of cancerous and non-cancerous cells following treatment with different formulations. (**A**) Untreated Colo-205 control cells displaying normal morphology with dense and well-organized cellular architecture. (**B**) Colo-205 cells treated with free curcumin, showing moderate morphological alterations, including partial cell rounding and reduced adherence. (**C**) Colo-205 cells treated with free piperine, showing mild to moderate morphological changes. (**D**) Colo-205 cells treated with AC6, exhibiting pronounced morphological alterations, including cell rounding, shrinkage, and partial loss of cellular integrity. (**E**) Colo-205 cells treated with 5-fluorouracil, showing extensive cellular disruption and loss of monolayer integrity. (**F**) Untreated CCD 841 control cells displaying normal epithelial morphology. (**G**) CCD 841 cells treated with free curcumin, showing minimal morphological alterations. (**H**) CCD 841 cells treated with free piperine and AC6, showing largely preserved cellular morphology with only minor changes. Scale bar: 200 µm.

Microscopic evaluation provided qualitative insight into the cellular responses of Colo-205 and CCD 841 cells following treatment. As shown in Figure 6, untreated Colo-205 control cells exhibited normal morphology, characterized by a dense and well-organized cellular architecture. In contrast, Colo-205 cells treated with the nanoparticle formulation AC6 showed evident morphological alterations, including cell rounding, reduced adherence, shrinkage, and partial loss of cellular integrity. These changes indicate treatment-induced cytotoxic stress and are consistent with the reduction in cell viability observed in the SRB assay.

The morphological alterations observed in AC6-treated Colo-205 cells were less pronounced than those induced by the reference drug 5-fluorouracil, which caused extensive cellular disruption and loss of monolayer integrity, consistent with its higher cytotoxic potency. Overall, the microscopic observations qualitatively support the SRB cytotoxicity results and confirm the anticancer activity of the curcumin-loaded BSA nanoparticle formulation under the tested conditions.

Microscopic evaluation of the non-cancerous CCD 841 cells further supported the quantitative viability findings. Untreated CCD 841 control cells displayed normal epithelial morphology with intact cellular architecture. Cells treated with free curcumin, free piperine, and AC6 showed only mild morphological alterations, with most cells retaining their structural integrity. In contrast to the pronounced cytotoxic effects observed in Colo-205 cancer cells, CCD 841 cells maintained their overall morphology after treatment, indicating limited cytotoxic stress. These observations are consistent with the high cell viability values obtained from the SRB assay and suggest a favorable selectivity profile for AC6 toward cancer cells over normal colon epithelial cells.

## 3. Discussion

Curcumin is a weakly acidic polyphenolic compound whose solubility and ionization behavior are highly dependent on the surrounding pH. Under acidic conditions, curcumin predominantly exists in a non-ionized form, which contributes to its extremely poor aqueous solubility [19]. Additionally, exposure to low pH may partially alter the conformation of the albumin matrix, potentially restricting matrix relaxation and limiting drug diffusion from the nanoparticles. These combined factors likely explain the relatively lower solubility enhancement observed at acidic pH. As the pH approaches neutral and mildly alkaline conditions, curcumin undergoes progressive deprotonation of its phenolic groups, resulting in the formation of more water-soluble ionized species (curcuminates). This pH-dependent ionization enhances aqueous dispersion and apparent solubility. The observed increase likely reflects a combination of molecular solubilization and colloidal dispersion of curcumin within the albumin nanoparticle system, rather than true thermodynamic solubility alone.

Concurrently, albumin nanoparticles may exhibit increased structural flexibility at neutral to slightly alkaline pH, promoting greater exposure of the encapsulated drug to the dissolution medium.

Beyond ionization effects, nanoscale formulation contributes independently to improved solubility. The high surface area-to-volume ratio of nanoparticles increases interfacial contact between curcumin and the surrounding medium, enhancing wettability and dissolution. Moreover, albumin nanoparticles help maintain curcumin in a dispersed state, minimizing aggregation and recrystallization. This stabilization likely plays a key role in the substantial solubility enhancement observed across the tested media.

The substantial increase in apparent solubility observed for the nanoparticle formulations can be attributed to a combination of improved molecular dispersion and stabilization of curcumin within a colloidal nanoparticulate system. Such nanoparticle-mediated dispersion enhances the apparent solubility of poorly water-soluble compounds by maintaining them in a dispersed state and preventing precipitation. In addition, pH-dependent ionization of curcumin, along with favorable structural behavior of the albumin matrix under neutral to mildly alkaline conditions, further contributes to the enhanced solubility. Overall, the improved solubility of curcumin from Cu-BSA-NPs appears to result from the combined effects of nanoscale dispersion, matrix-mediated stabilization, and pH-dependent behavior.

Overall, the improved solubility of curcumin from Cu-BSA-NPs appears to result from the combined influence of pH-dependent ionization, favorable matrix behavior at neutral to mildly alkaline conditions, and nanoscale dispersion effects.

Nanoparticles within the 100–150 nm size range have been widely reported to favor cellular uptake in in vitro cancer models, particularly via endocytic pathways. However, no in vivo biodistribution, accumulation, or enhanced permeation and retention (EPR)-related behavior was evaluated in the present study [20,21].

The smaller particle sizes observed in batches AC1-AC3, combined with higher PDI values, suggest a heterogeneous particle population, potentially arising from aggregation or less controlled synthesis conditions. In contrast, batches AC4-AC7 demonstrated moderately increased particle size with reduced PDI values, indicating improved uniformity and better formulation control.

Zeta potential measurements showed a progressive shift toward more negative values across formulations. This likely reflects greater surface exposure of ionizable functional groups from albumin and curcumin, enhancing electrostatic repulsion and colloidal stability. The zeta potential of approximately −30 ± 2.0 mV observed for AC6 falls within a range generally associated with stable nanoparticle dispersions under experimental conditions.

The relatively narrow size distribution and favorable surface charge of AC6 support its suitability for in vitro cellular interaction studies. The particle sizes obtained in the present study (96.7 ± 10.5–126.4 ± 35.8 nm) fall within the nanoscale range commonly investigated for in vitro nanoparticle interaction studies. Particle size alone is not sufficient to achieve colon-specific targeting. In the present formulation, the observed pH-dependent differences in release behavior are primarily attributed to the pH-dependent nature of the albumin matrix, which remains relatively compact under acidic conditions and facilitates drug release at neutral to slightly alkaline pH.

Albumin-binding proteins such as gp60 and SPARC have been reported in the literature to facilitate albumin transport across endothelial barriers. However, receptor-mediated uptake was not experimentally investigated in the present study and is discussed here only in the context of previously reported findings [9,10].

Entrapment efficiency was strongly influenced by the interplay between protein content, drug concentration, and crosslinking density. The highest entrapment efficiency observed at a 2:1 BSA:Curcumin ratio (AC6) likely reflects an optimal balance between sufficient carrier matrix and manageable drug loading. The variation in entrapment efficiency observed among the formulations can be attributed to differences in albumin concentration, drug loading, and crosslinker level. Higher BSA concentrations provide additional binding sites and matrix volume for drug encapsulation, whereas excessive crosslinking may produce a denser protein network that restricts drug accommodation and lowers entrapment efficiency.

While crosslinking is essential for nanoparticle stabilization, excessive crosslinker concentration (EDC ≥ 1.6 mg) likely produces a rigid, densely packed network that restricts internal free volume and limits drug accommodation. Conversely, lower crosslinker levels may yield a more flexible matrix with greater accessible interaction domains, improving encapsulation efficiency.

Particle size was also influenced by formulation composition. Increased albumin concentration may promote intermolecular interactions and aggregation, resulting in larger particle dimensions. Thermal analysis further supports structural changes in albumin upon heating. As temperature rises, non-covalent interactions stabilizing protein structure are disrupted, leading to aggregation. BSA contains three domains that can denature independently, which explains the multiple transition peaks observed in DSC rather than a single sharp melting peak.

The FTIR and DSC findings collectively provide insight into the physicochemical state of curcumin within the nanoparticle system. FTIR analysis confirmed the retention of characteristic functional groups along with evidence of crosslinking within the albumin matrix, indicating successful incorporation without chemical degradation. The DSC thermogram of the loaded nanoparticles showed a shift and slight broadening of the characteristic melting peak of curcumin, suggesting reduced crystallinity and partial amorphization of the drug within the BSA matrix [22].

Such transformation from a crystalline to a more amorphous or molecularly dispersed state is known to enhance the apparent solubility and dissolution behavior of poorly water-soluble drugs. In addition, the interaction of curcumin with the protein matrix may contribute to its stabilization in a dispersed form, which can facilitate diffusion during drug release. These physicochemical changes are therefore consistent with the improved solubility observed in the present study.

SEM analysis confirmed that Cu-BSA-NPs were discrete, spherical, and smooth in morphology. Albumin’s globular structure naturally favors spherical assembly. EDC-mediated crosslinking stabilizes this structure through amide bond formation between carboxyl and amine groups without significantly altering surface morphology [11,23].

The in vitro drug release profile demonstrated pH-dependent differences in release behavior, with comparatively minimal release under acidic and near-neutral conditions and enhanced release at pH 7.4. These findings indicate that release behavior was influenced by environmental pH under the tested in vitro conditions rather than confirmed colon-targeted delivery.

AC6 exhibited the highest cumulative release at pH 7.4, likely due to its optimized particle size, improved solubility, and higher entrapment efficiency. Bovine serum albumin contains multiple ionizable amino acid residues that can undergo conformational changes depending on the surrounding pH. Under acidic conditions, albumin tends to maintain a relatively compact structure, which may restrict drug diffusion from the nanoparticle matrix. At near-neutral or slightly alkaline pH, partial unfolding or expansion of the protein network can occur, increasing matrix permeability and facilitating drug release [24,25,26].

EDC acts as a zero-length crosslinking agent that promotes the formation of amide bonds between carboxyl and amine groups within the albumin structure [27]. This crosslinking stabilizes the nanoparticle network while preserving the inherent pH-dependent conformational flexibility of the protein matrix, which may contribute to the observed pH-dependent release behavior. While EDC-mediated crosslinking plays an important role in stabilizing the nanoparticle structure and modulating drug release, its use also warrants consideration from a safety perspective. During nanoparticle preparation, unreacted EDC is expected to be removed through purification steps such as repeated washing and centrifugation. Future studies should evaluate residual reagent levels and their potential biological implications to ensure formulation safety for in vivo applications.

The role of crosslinking in nanoparticle formation, discussed in relation to entrapment efficiency, also appears to influence drug release behavior. EDC-mediated crosslinking forms a three-dimensional protein network, and the extent of this crosslinking determines how compact or permeable the matrix becomes. In the optimized formulation (AC6), the relatively lower crosslinking level likely results in a more flexible structure, allowing easier diffusion of the drug. In contrast, higher crosslinker concentrations may create a tighter and more rigid network that restricts drug movement.

This structural difference helps explain the observed release pattern, where enhanced drug release at pH 7.4 may be associated with increased matrix relaxation and diffusion under near-neutral conditions. In turn, this improved drug availability may contribute to the enhanced cytotoxic response observed for the optimized formulation.

Curcumin’s own pH-dependent solubility further contributes to the release profile. At pH 7.4, partial ionization enhances its diffusion once released from the matrix [28]. In contrast, limited solubility under acidic conditions restricts release. Thus, the combined pH-sensitive properties of both albumin and curcumin govern overall release behavior [29].

Formulations with lower cumulative release, particularly AC1-AC5, may reflect higher crosslinking density or suboptimal carrier-to-drug ratios, which reduce matrix permeability and limit diffusion [30].

The strong correlation with the Higuchi model (R^2^ = 0.9543) indicates diffusion-controlled release from a matrix system [31]. The Korsmeyer–Peppas exponent (*n* = 0.241) confirms a Fickian diffusion mechanism, suggesting that drug release occurred primarily through diffusion rather than matrix erosion or swelling [32]. The albumin matrix likely acted as a diffusion barrier, enabling sustained release over time [33].

The cytotoxic response observed in Colo-205 cells exhibited a concentration-dependent but non-linear pattern, particularly at higher concentrations. This behavior may be associated with saturation-related effects and partial nanoparticle aggregation at elevated concentrations, which could limit proportional increases in cytotoxic response [30].

Comparative evaluation with free curcumin and free piperine demonstrated that the nanoparticle formulation AC6 exhibited enhanced cytotoxic activity. AC6 showed a lower IC_50_ value than both free curcumin and free piperine, together with greater growth inhibition at equivalent concentrations, indicating enhanced in vitro cytotoxic activity under the tested conditions.

Overall, these results suggest that nanoparticles enhanced the in vitro anticancer activity of curcumin. This effect is likely associated with improved dispersibility, stabilization of curcumin in the culture medium, and sustained availability under in vitro conditions. Although albumin-binding proteins, such as gp60 and SPARC, have been reported to facilitate albumin transport in tumor tissues [12], receptor-mediated uptake mechanisms were not investigated in the present study. Therefore, the observed cytotoxic effects should be interpreted primarily in terms of improved physicochemical and biopharmaceutical behavior rather than active targeting.

Although both curcumin and piperine possess intrinsic anticancer properties, the free compounds exhibited comparatively lower cytotoxic effects under identical experimental conditions. Piperine is also known to act as a bioavailability enhancer; however, its specific contribution within the nanoparticle system was not independently evaluated in the present study. Accordingly, no conclusions regarding synergistic interactions can be drawn [34,35]. Furthermore, because a control nanoparticle formulation without piperine was not investigated, the individual contribution of piperine to the observed cytotoxicity and release behavior cannot be independently distinguished from the effects associated with nanoparticle-mediated curcumin delivery alone. Bovine serum albumin nanoparticles have been widely investigated as biocompatible carriers capable of improving solubility and enabling controlled release [24,25]. In the present work, encapsulation of curcumin within the BSA matrix appears to improve its apparent availability, contributing to the observed cytotoxic response.

The concentrations used in the cytotoxicity study were selected to establish a complete dose–response profile and determine IC_50_ values; however, these levels may not directly correspond to achievable in vivo concentrations. Therefore, the results should be interpreted as indicative of relative cytotoxic potential rather than absolute therapeutic efficacy.

The cytotoxicity evaluation in the non-cancerous CCD 841 cell line provides important insight into the selectivity and preliminary safety profile of the developed formulation. All tested samples, including the nanoparticle formulation, exhibited minimal cytotoxic effects on normal colon epithelial cells, with cell viability remaining above 90% across the tested concentration range.

Notably, although the nanoparticle formulation showed improved cytotoxic activity against Colo-205 cancer cells, a comparable reduction in viability was not observed in normal epithelial cells. This differential response suggests a degree of selective cytotoxicity, which may be attributed to differences in cellular susceptibility with the nanoparticulate system under in vitro conditions.

These findings support the potential advantage of the formulation in enhancing anticancer activity while maintaining acceptable compatibility with normal cells. However, further in vivo studies are required to confirm this selectivity and to establish the safety profile of the formulation under physiological conditions.

Overall, these findings demonstrate that Cu-BSA-NPs exhibit measurable in vitro anticancer activity. While the results highlight the potential of albumin-based nanocarriers for delivering poorly soluble anticancer agents, further studies are required to clarify uptake mechanisms, intracellular fate, and in vivo therapeutic relevance.

## 4. Materials and Methods

Bovine serum albumin (BSA), piperine, and 1-ethyl-3-(3-dimethylaminopropyl) carbodiimide hydrochloride (EDC·HCl) were purchased from LOBA Chemie Pvt. Ltd., Mumbai, India. Curcumin was kindly provided as a gift sample by Bio-Med Ingredients, Goa, India. All other chemicals and reagents were obtained from local suppliers and were of analytical grade. The human colorectal adenocarcinoma cell line Colo-205 was obtained from the National Centre for Cell Sciences (NCCS), Pune, India. The cells were maintained at 37 °C in a humidified incubator with 5% CO_2_ and balanced air. Cell culture was carried out in Dulbecco’s Modified Eagle Medium (DMEM) supplemented with 10% fetal bovine serum.

### 4.1. Formulation of Cu-BSA-NPs (Cu-BSA-NPs)

Cu-BSA-NPs were prepared using a controlled desolvation technique under optimized processing conditions to ensure reproducibility and consistent particle characteristics [17]. Bovine serum albumin was dissolved in 3.0 mL of distilled water, and the pH of the solution was adjusted to 7.4 ± 0.1 prior to desolvation to maintain optimal albumin conformation for nanoparticle formation. Curcumin and piperine were co-dissolved in 8.0 mL of ethanol and added dropwise to the aqueous BSA solution at a controlled rate of 1.0 mL/min under continuous magnetic stirring (500 rpm) at room temperature. The resulting desolvated albumin nanoparticles were subsequently stabilized by chemical crosslinking using 1-ethyl-3-(3-dimethylaminopropyl) carbodiimide hydrochloride (EDC·HCl). For the preparation of different formulations, varying amounts of EDC·HCl were dissolved in a fixed volume of 0.5 mL of distilled water to obtain different crosslinker concentrations across batches, as detailed in Table 2. The freshly prepared EDC solution was added to the nanoparticle dispersion under continuous stirring, and the crosslinking reaction was allowed to proceed for 24 h at room temperature to enhance the structural stability of the nanoparticle matrix. The nanoparticles were subsequently purified by five cycles of differential centrifugation at 12,000 rpm for 8 min. Following each centrifugation step, the supernatant containing residual crosslinker, unencapsulated drug, and soluble albumin was carefully removed. The resulting nanoparticle pellet was then redispersed in distilled water using mild sonication for 5 min before proceeding to the next purification cycle [36].

### 4.2. Characterization of Cu-BSA-NPs

#### Apparent Solubility Study Top of Form

An excess amount of nanoparticles (equivalent to 100 mg NPs/mL) was dispersed in 5.0 mL of distilled water, 0.1 N HCl (pH 1.2), phosphate buffer (pH 6.8), and phosphate buffer (pH 7.4) in separate glass vials. The suspensions were agitated for 48 h to ensure equilibrium solubility. After equilibration, the samples were filtered through Whatman No. 42 filter paper and analyzed spectrophotometrically at 426 nm using a double-beam UV-Visible spectrophotometer (Model UV-2401 (PC), Shimadzu, Kyoto, Japan). The apparent solubility of plain curcumin was also determined under identical conditions in the respective media for comparison. A standard calibration curve for curcumin was constructed over the concentration range of 2–10 µg/mL, demonstrating compliance with Beer–Lambert’s law (R^2^ = 0.9888).

### 4.3. Drug Loading Efficiency

An accurately weighed quantity of Cu-BSA-NPs (100 mg) was dispersed in 5.0 mL of ethanol and subjected to continuous magnetic stirring for 24 h to ensure complete drug extraction. The dispersion was then centrifuged at 4000 rpm for 15 min, and the supernatant was collected for analysis. The drug content was quantified spectrophotometrically at 426 nm using a double-beam UV-Visible spectrophotometer [37].

### 4.4. Particle Diameter and Zeta Potential

The particle size, zeta potential, and polydispersity index (PDI) of Cu-BSA-NPs were determined using a HORIBA SZ-100 instrument (HORIBA Ltd., Kyoto, Japan) at 25 °C. Prior to analysis, the samples were allowed to equilibrate overnight at room temperature to ensure stability and were gently inverted several times to achieve uniform dispersion. Each measurement was performed in triplicate, and the reported values represent the mean of twenty individual readings [37].

### 4.5. Differential Scanning Calorimetry (DSC)

The thermal behavior of curcumin, unloaded nanoparticles, and curcumin-loaded nanoparticles was evaluated using differential scanning calorimetry (DSC) (Model DSC 620, Seiko Nanotechnology, Japan). Thermal analysis was performed over a temperature range of 50 °C to 450 °C at a heating rate of 10 °C/min in aluminum pans under a nitrogen atmosphere maintained at a constant flow rate of 20 mL/min [38].

### 4.6. Scanning Electron Microscopy (SEM)

The surface morphology of the nanoparticles was examined using a scanning electron microscope (S-3700N, Hitachi, Ibaraki, Japan). Prior to analysis, the samples were vacuum-dried in a desiccator to remove residual moisture. The dried nanoparticles were then mounted onto a sample stub, and micrographs were acquired to evaluate particle shape and surface characteristics [39].

### 4.7. Fourier-Transform Infrared Spectroscopy (FTIR)

The Fourier-transform infrared (FTIR) spectra of curcumin, BSA, the crosslinker (EDC·HCl), their physical mixture, and Cu-BSA-NPs were recorded over the wavenumber range of 4000–400 cm^−1^ using the potassium bromide (KBr) pellet method. Spectral analysis was performed with an FTIR spectrometer (Model 8400S, Shimadzu Asia Pacific Pvt. Ltd., Singapore) [40].

### 4.8. In Vitro Drug Release Study

The in vitro release profile of curcumin from Cu-BSA-NPs was evaluated using a USP Type II (paddle) dissolution apparatus. The dissolution study was performed sequentially in different media to simulate gastrointestinal conditions. Initially, the nanoparticles were exposed to 250.0 mL of dissolution medium at pH 1.2 for 2 h, followed by replacement with phosphate buffer (pH 6.8) for 4 h, and subsequently continued in phosphate buffer (pH 7.4) for an additional 12 h. An activated dialysis membrane containing Cu-BSA-NPs (equivalent to 500 mg of curcumin) suspended in 5 mL of distilled water was securely attached to the paddle assembly and immersed in the dissolution medium. At predetermined time intervals, 5.0 mL aliquots were withdrawn and analyzed spectrophotometrically at 426 nm. Each withdrawn volume was immediately replaced with an equal volume of fresh, drug-free dissolution medium to maintain sink conditions [41,42]. The cumulative release data were further analyzed by fitting to various kinetic models, including zero-order, first-order, Higuchi, and Korsmeyer–Peppas equations, to elucidate the underlying drug release mechanisms.

### 4.9. Stability Study

Stability studies were conducted at 25 ± 2 °C and 60 ± 5% relative humidity in accordance with ICH Q1A(R2) guidelines for long-term storage conditions.

Cu-BSA-NPs (AC6) were stored for six months under controlled conditions at 25 ± 2 °C and 60 ± 5% relative humidity. At predetermined intervals, the samples were visually inspected for any signs of physical instability, such as aggregation or discoloration, and evaluated for changes in drug loading efficiency [43].

### 4.10. Colo-205 Cell Line Study

The human colon cancer cell lines Colo-205 were procured and evaluated at Tata Memorial Centre–Advanced Centre for Treatment, Research and Education in Cancer (ACTREC), Mumbai, India, a recognized national cancer research facility.

The Colo-205 cells were maintained in complete growth medium supplemented with 10% fetal bovine serum and 2 mM L-glutamine under standard cell culture conditions. Cells were seeded in 96-well microtiter plates at a density of 5000 cells/well in a total volume of 100 µL and incubated at 37 °C in a humidified atmosphere containing 5% CO_2_ for 24 h before treatment.

The cytotoxic effects of Cu-BSA-NPs, free curcumin, free piperine, and the standard anticancer drug 5-fluorouracil were evaluated using the sulforhodamine B (SRB) assay. Stock solutions or suspensions of the test samples were prepared in sterile distilled water. The nanoparticle suspension was thawed before use and diluted to obtain an initial concentration of 1 mg/mL, expressed as curcumin equivalent. An aliquot of 10 µL of each working solution was added to wells containing 90 µL of culture medium to achieve final concentrations of 10, 20, 40, 60, 80, 100, and 200 µg/mL. Following treatment, the plates were incubated under standard cell culture conditions for 48 h. At the end of the incubation period, the assay was terminated by adding cold trichloroacetic acid (TCA) to fix the cells, followed by incubation at 4 °C for 60 min. The supernatant was then carefully removed, and the plates were washed and air-dried. Fixed cells were stained with 0.4% (*w*/*v*) sulforhodamine B solution for 30 min at room temperature. Unbound dye was removed by washing with 1% acetic acid, and the plates were air-dried. The protein-bound dye was subsequently solubilized using Tris base solution, and absorbance was measured at 540 nm with a reference wavelength of 690 nm using a microplate reader [44]. Cell viability was calculated by comparing the mean absorbance of treated wells with that of untreated control wells, and percentage inhibition was determined accordingly [45]. Percentage viability was calculated using the following equation:

$$\mathrm{Percent}\;\mathrm{Viability}\;=\;\left(\frac{Average\;absorbance\;of\;the\;test\;wells\;}{Average\;absorbance\;of\;the\;control\;wells}\right)\times 100$$

Dose–response curves were generated from the SRB assay data, and IC_50_ values were calculated using nonlinear regression analysis.

To assess the safety and selectivity of the developed formulation, cytotoxicity was also evaluated in the non-cancerous human colon epithelial cell line CCD 841 using the SRB assay. Cells were cultured in DMEM supplemented with fetal bovine serum and antibiotic-antimycotic solution under standard culture conditions. CCD 841 cells were treated with AC6, free curcumin, free piperine, and 5-fluorouracil at concentrations ranging from 10 to 200 µg/mL for 48 h. Following treatment, cell viability was determined using the SRB assay as described above, and percentage viability was calculated relative to untreated control cells.

## 5. Statistical Analysis

All experiments were performed in triplicate, and results are expressed as mean ± standard deviation (SD). Statistical analysis was performed using one-way analysis of variance (ANOVA), followed by Tukey’s post hoc test to determine significant differences between groups. Differences were considered statistically significant at *p* < 0.05.

## 6. Conclusions

The present study reports the successful formulation and systematic in vitro evaluation of bovine serum albumin (BSA)-based curcumin nanoparticles designed to enhance curcumin solubility and enable controlled drug release. Multiple formulations were developed and characterized, among which batch AC6 demonstrated the most favorable physicochemical profile, including improved aqueous solubility, nanoscale particle size with acceptable distribution, higher entrapment efficiency, and reproducible pH-dependent release behavior.

Sequential pH-dependent release studies revealed minimal drug release under acidic (pH 1.2) and near-neutral (pH 6.8) conditions, followed by an enhanced increase at pH 7.4, indicating that release is governed by matrix behavior and formulation composition under alkaline environments. Evaluation of cytotoxic activity in the Colo-205 cell line revealed that treatment with AC6 resulted in a concentration-dependent reduction in cell viability. Microscopic analysis further confirmed morphological changes consistent with cytotoxic stress in treated cells compared with untreated controls.

The observed anticancer effects are likely associated with improved curcumin dispersion, stabilization within the nanoparticle system, and extended availability under in vitro conditions. Collectively, these findings suggest that Cu-BSA-NPs represent a biocompatible nanoparticulate system capable of improving curcumin dispersibility and exhibiting measurable in vitro anticancer activity.

However, these findings are based on in vitro evaluation, and comprehensive in vivo studies, including pharmacokinetic profiling, biodistribution analysis, and safety assessment, are required to establish therapeutic relevance and translational potential.

## Figures and Tables

**Table 1:** Apparent solubility of Cu-BSA-NPs (AC1–AC7) in different media.

S. No.	Batches	Apparent Solubility of Cu-BSA-NPs (mg/mL)				Solubility Enhancement Ratios			
		Water	pH 1.2	pH 6.8	pH 7.4	Water	pH 1.2	pH 6.8	pH 7.4
1	Curcumin	0.00027 ± 0.0001	0.000153 ± 0.00002	0.00018 ± 0.0001	0.00029 ± 0.0001				
2	AC1	0.0084 ± 0.0011 ^ns^	0.00173 ± 0.0002 *	0.00256 ± 0.0007 *	0.0189 ± 0.0019 ^ns^	31.11 ± 12.04	11.31 ± 1.33	14.22 ± 1.12	65.17 ± 19.49
3	AC2	0.0375 ± 0.0046 ^ns^	0.00427 ± 0.0004 ***	0.0034 ± 0.0008 ***	0.0337 ± 0.0076 **	138.88 ± 11.91	27.91 ± 2.69	18.88 ± 10.26	116.20 ± 42.39
4	AC3	0.0156 ± 0.0067 *	0.00237 ± 0.001 **	0.00266 ± 0.0009 **	0.0203 ± 0.0081 ^ns^	57.77 ± 25.86	15.49 ± 4.79	14.77 ± 6.29	70.0 ± 32.46
5	AC4	0.0315 ± 0.0053 **	0.00303 ± 0.00025 ***	0.0045 ± 0.0006 ***	0.046 ± 0.0125 ***	116.66 ± 33.29	19.80 ± 4.92	25.0 ± 4.15	158.62 ± 38.88
6	AC5	0.0186 ± 0.0124 ^ns^	0.00327 ± 0.00046 ***	0.00386 ± 0.0006 ***	0.0377 ± 0.0065 ***	68.88 ± 41.17	21.37 ± 2.27	21.44 ± 3.24	130.0 ± 4.58
7	AC6	0.0757 ± 0.0085 ***	0.00803 ± 0.0005 ***	0.0093 ± 0.0006 ***	0.0942 ± 0.0045 ***	280.37 ± 62.86	52.48 ± 5.31	51.66 ± 12.48	324.83 ± 57.79
8	AC7	0.0278 ± 0.0065 ^ns^	0.0038 ± 0.00071 ***	0.00453 ± 0.0012 ***	0.0363 ± 0.0111 ***	102.96 ± 40.63	24.83 ± 4.55	25.16 ± 1.53	125.17 ± 14.71

Values represent mean ± SD (*n* = 3). Statistical analysis was performed using one-way ANOVA followed by Tukey–Kramer multiple comparison test. * *p* < 0.05, ** *p* < 0.01, *** *p* < 0.001, ^ns^ = non-significant compared with native curcumin.

**Table 2:** Formulation composition, drug loading efficiency, and entrapment efficiency of Cu-BSA-NPs.

Batch Code	Bovine Serum Albumin (mg)	Curcumin (mg)	Piperine (mg)	1-(3-Dimethylamineopropyl)-3-Ethylcarbodimide Hydrochloride (mg) *	% Curcumin Loading Efficiency	% Entrapment Efficiency
AC1	200	50	20	5	1.308 ± 0.206	6.59 ±1.11
AC 2	400	200	20	1.6	11.093 ± 0.141	35.11 ±0.603
AC 3	400	400	20	1.6	8.926 ± 0.341	18.71 ± 0.713
AC 4	400	200	20	1.25	10.584 ± 0.702	33.53 ± 2.92
AC 5	400	400	20	1.25	9.629 ± 0.555	20.22 ± 1.31
AC 6	400	200	20	0.5	16.744 ± 0.266	52.98 ± 0.65
AC 7	400	400	20	0.5	12.946 ± 0.573	27.19 ± 1.67

Values are expressed as mean ± SD (*n* = 3). EDC·HCl was used as a crosslinking agent. Piperine concentration was kept constant across all formulations. * For each formulation, the indicated quantity of EDC·HCl was prepared in 0.5 mL of distilled water.

**Table 3:** Physicochemical Properties of Cu-BSA-NPs, Including Particle Size, Polydispersity Index, and Zeta Potential.

S. No.	Batch	Particle Size (nm)	PDI	Zeta Potential (mV)
1	AC1	96.7 ± 10.5	0.581	−18.2 ± 1.01
2	AC2	108.3 ± 26.2	0.256	−22.2 ± 1.0
3	AC3	109.8 ± 40.9	0.352	−22.7 ± 2.3
4	AC4	111.8± 33.5	0.325	−25 ± 3.1
5	AC5	115.9 ± 60.1	0.377	−27.7 ± 2.5
6	AC6	118.4 ± 29.6	0.289	−30 ± 2.0
7	AC7	126.4 ± 35.8	0.456	−34 ± 1.0

Data are expressed as mean ± SD (*n* = 3).

**Table 4:** Release kinetic modeling parameters of formulation AC6.

Kinetic Model	R^2^ Value	Slope	Intercept
Zero-order	0.8481	0.0934	39.7112
First-order	0.5822	0.0013	1.2719
Hixson–Crowell	0.6990	0.0077	5.4210
Korsmeyer–Peppas	0.9547	0.2410	1.5919
Higuchi	0.9543	0.2895	−3.8062

Values represent regression parameters obtained from fitting the in vitro release data of formulation AC6 to different kinetic release models.

**Table 5:** Stability assessment of Cu-BSA-NPs (AC6) stored at 25 ± 2 °C and 60 ± 5% relative humidity (RH).

**Time (Months)**	**Physical Appearance**	**Drug Loading Efficiency (%)**	**Entrapment Efficiency (%)**	**Particle Size (nm)**	**PDI**	**Zeta Potential (mV)**
0.	No visible physical changes	16.77 ± 0.61	52.00 ± 1.90	118.4 ± 29.6	0.289	−30 ± 1
1.	No visible physical changes	16.63 ± 0.62	51.56 ± 1.91	108.3 ± 25.6	0.256	−31.7 ± 1.9
3.	No visible physical changes	16.22 ± 0.60	50.27 ± 1.87	122.4 ± 10.7	0.215	−31.5 ± 1.7
6.	No visible physical changes	15.93 ± 0.52	49.37 ± 1.60	120.4 ± 19.6	0.257	−32 ± 2

Values are expressed as mean ± SD (*n* = 3). Statistical analysis was performed using Student’s *t*-test by comparing each time point with initial values (0 month). No statistically significant differences were observed (*p* > 0.05).

## Data Availability

The original contributions presented in this study are included in the article/Supplementary Materials. Further inquiries can be directed to the corresponding author(s).

## Supplementary Materials

The following supporting information can be downloaded at: https://www.mdpi.com/article/10.3390/ph19060872/s1. Data for Colo-205 Human Cell Line Study, IC50 calculation, Data for the effect against CCD 841 Cell lines.
